# Leptomeningeal Carcinomatosis from Squamous Cell Carcinoma of the Ethmoid Sinus: A Case Report

**DOI:** 10.7759/cureus.5281

**Published:** 2019-07-30

**Authors:** Samer G Zammar, Max Hennessy, Joshua Warrick, Neerav Goyal, Brad E Zacharia

**Affiliations:** 1 Neurosurgery, Penn State Health Milton S. Hershey Medical Center, Hershey, USA; 2 Otolaryngology, Penn State Health Milton S. Hershey Medical Center, Hershey, USA; 3 Pathology, Penn State Health Milton S. Hershey Medical Center, Hershey, USA

**Keywords:** leptomeningeal carcinomatosis, sinonasal cancer, sinus, head, cerebrospinal fluid, metastasis

## Abstract

Leptomeningeal carcinomatosis (LMC) is an end-stage disease with poor prognosis. This disease has not yet been reported with sinonasal carcinomatosis. We present a case of a patient who presented with posterior ethmoid/anterior cranial mass which turned out to be poorly differentiated squamous cell carcinoma (SCC). Later the patient presented with enhancement of the spinal roots and a lumbar puncture diagnosed the leptomeningeal spread of her primary disease. After intrathecal chemotherapy and palliative radiation, the patient failed to resist her disease seven months after the diagnosis. We present the first case report of leptomeningeal spread of sinonasal cancer. Although it seems rare, LMC should be on the differential of patients presenting with neurological deficits.

## Introduction

Leptomeningeal carcinomatosis (LMC), or carcinomatosis meningitis, is the infiltration of neoplastic cells into the leptomeninges. LMC is a relatively common and lethal complication caused by a variety of cancers, and is reported to occur in 1%-8% of patients with malignancies [1]. LMC is most commonly a result of breast and lung cancer but can be caused by a variety of other solid tumors [2]. Sinonasal carcinomas are rare and aggressive tumors of the nasal passage and paranasal sinuses that account for only 3%-5% of all head and neck cancers [3-5]. Due to the rarity of sinonasal carcinomas, LMC caused by a sinonasal carcinoma is exceedingly rare. We present, to our knowledge, the first reported case of LMC from a sinonasal squamous cell carcinoma (SCC).

## Case presentation

**Figure 1:**
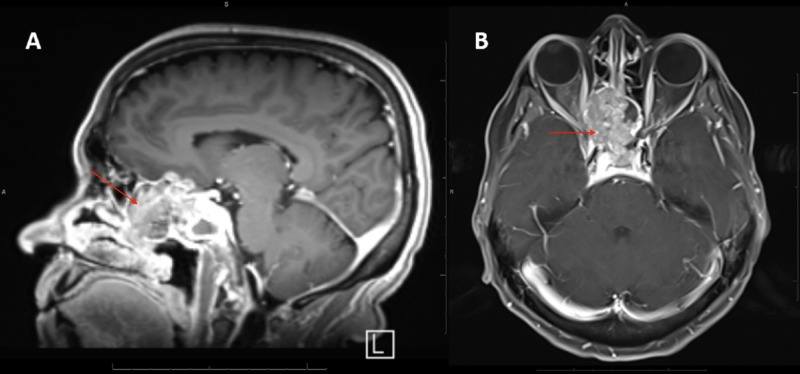
T1-enhanced magnetic resonance imaging (MRI) with fat suppression showing sinonasal squamous cell carcinoma (SCC) of the ethmoid air cells extending into the adjacent ethmoid sinus A: Sagittal view; B: Axial view.

A 66-year-old woman with a past medical history of hypertension presented with progressively worsening right eye visual acuity. Magnetic resonance imaging (MRI) revealed a large, heterogeneously enhancing mass centered in the right posterior ethmoid air cells, extending into the left ethmoid sinus and the right anterior cranial fossa, producing mass effect on the right optic canal and right optic nerve (Figure 1).

**Figure 2:**
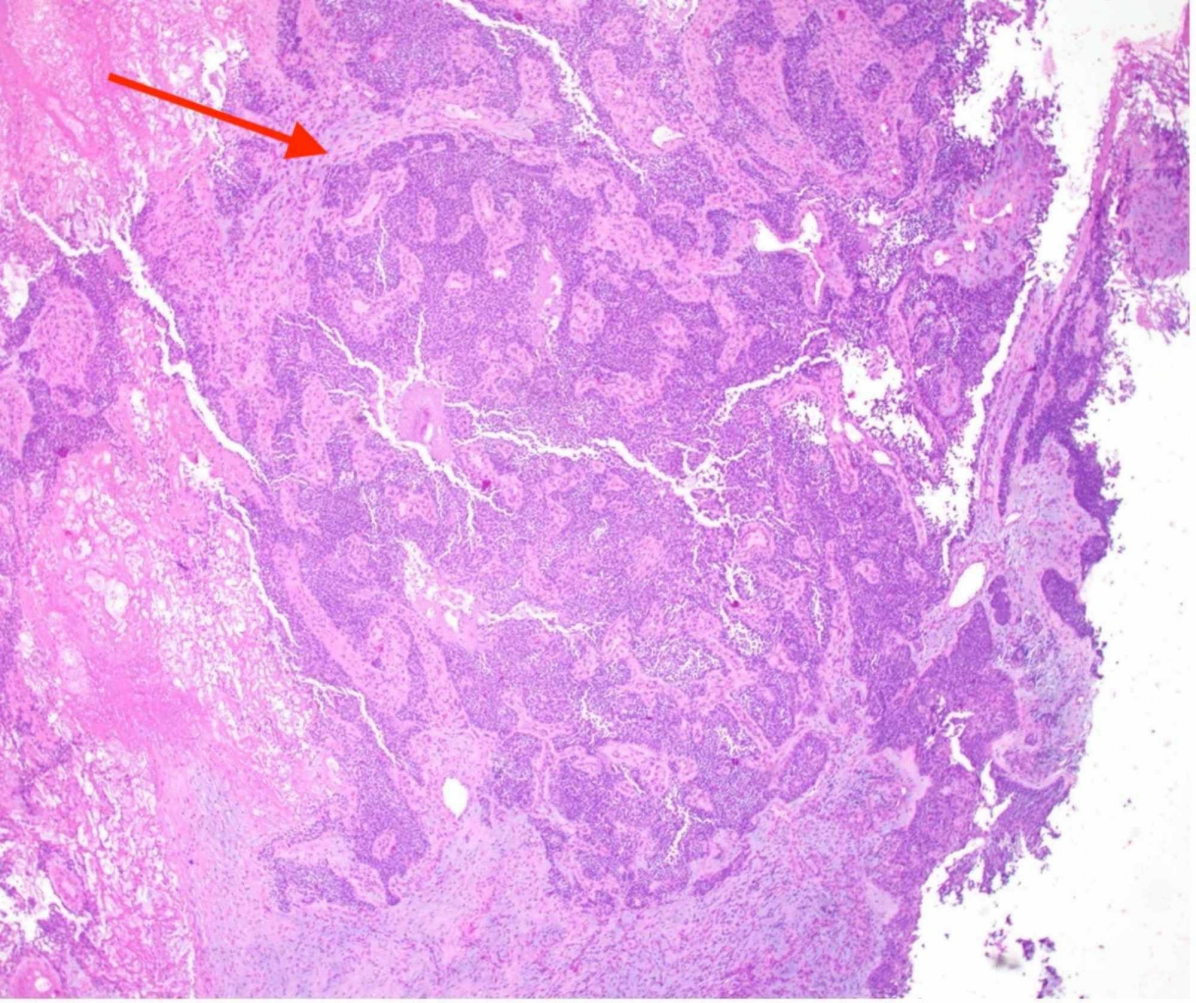
At low power (40x magnification), the tumor appears as invasive nests of cohesive tumor cells within a desmoplastic stroma

**Figure 3:**
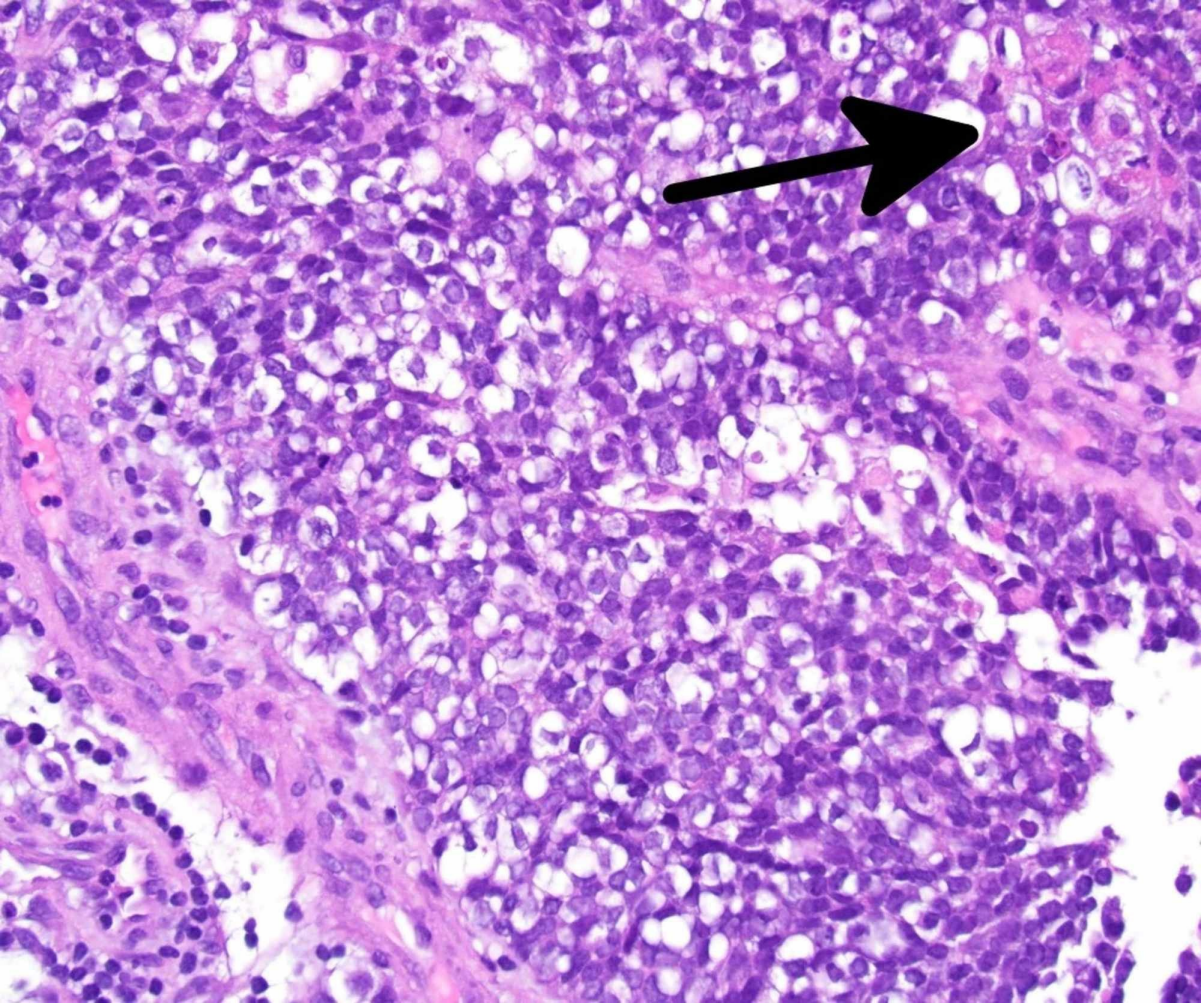
At high power (400x magnification), the tumor cells are seen to have large, hyperchromatic nuclei, as well as minimal cytoplasm, imparting a “basaloid” appearance. Intercellular bridges were present, as were foci of vague keratinization (arrow)

**Figure 4:**
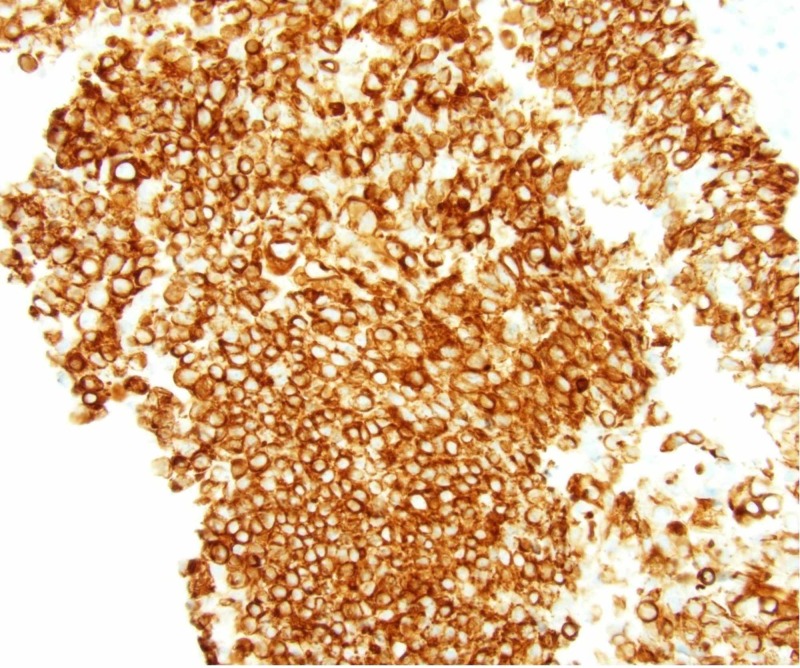
By immunohistochemistry, tumor cells were positive for cytokeratin 5/6, as seen in squamous cell carcinoma. Malignant cells were present in the cerebrospinal fluid specimen evaluated by cytology

An endoscopic endonasal biopsy demonstrated a poorly differentiated Epstein-Barr virus (EBV) negative carcinoma. Histologically, the tumor was interpreted as a poorly-differentiated, SCC of the ethmoid sinuses with basaloid features, T4aN0M0 (Stage IVa, (American Joint Committee on Cancer (AJCC) 7th edition) (Figures 2-4).

Positron emission tomography/computed tomography (PET/CT) performed 15 days after the biopsy did not show any features of nodal or distant metastases. The treatment plan consisted of neoadjuvant fractionated external beam radiation with weekly cisplatin to be followed by definitive surgical resection.

**Figure 5:**
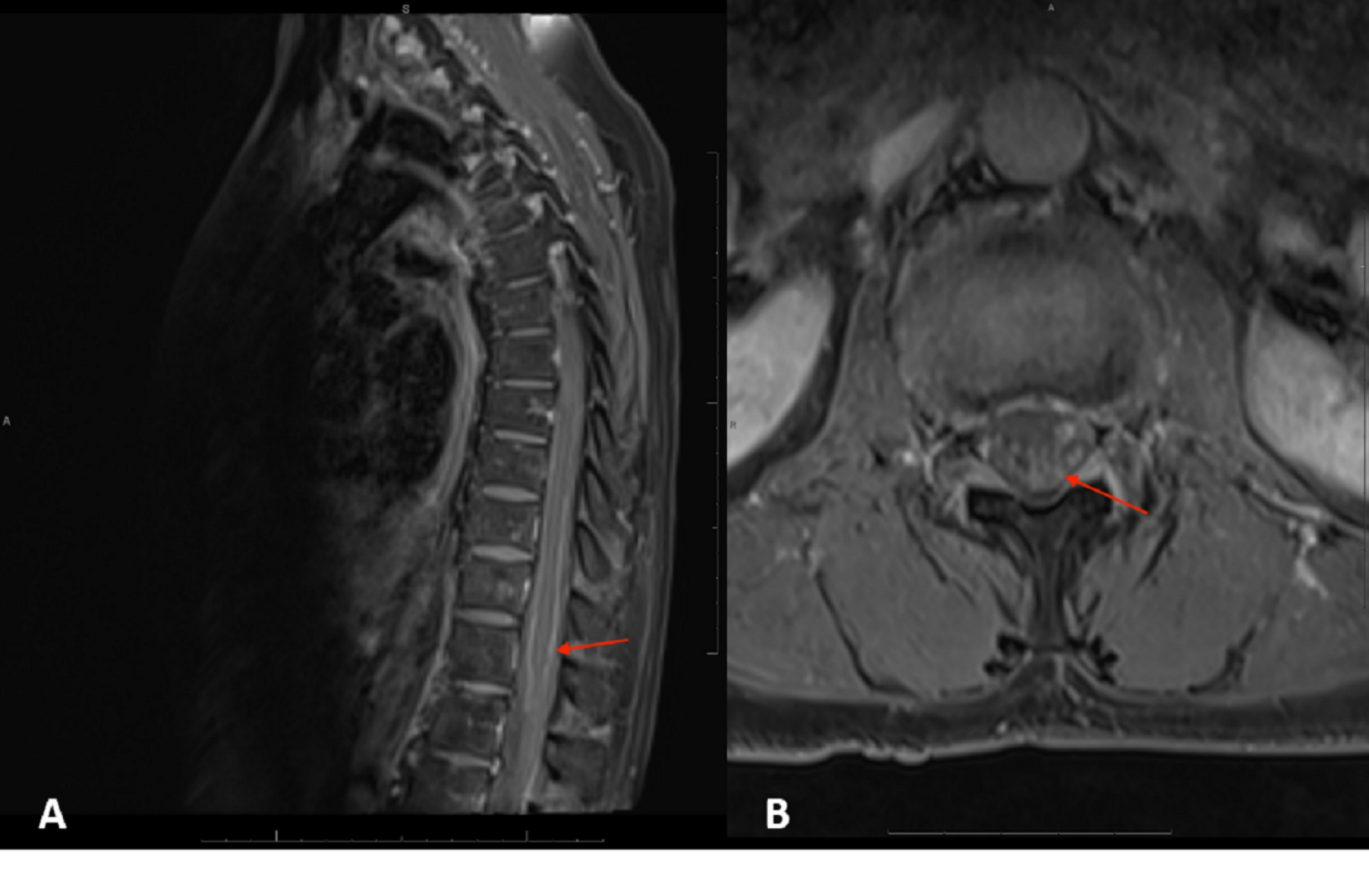
T1-weighted magnetic resonance imaging (MRI) with contrast and fat suppression of the spine that was interpreted as arachnoiditis (A) Sagittal view of lumbar spine; (B) L4-L5 nerve root clumping was the only suspicious sign for leptomeningeal carcinomatosis (LMC) on MRI

The tumor initially responded well to the neoadjuvant radiation and chemotherapy, and decreased in size, from 2.3 x 2.7 cm in greatest dimension to 1.4 x 1.8 cm. However, approximately one month after finishing chemoradiation, and just days before scheduled surgical resection, the patient presented to the emergency department (ED) for low back pain and weakness that radiated to the bilateral lower extremities. An MRI of the cervical, thoracic, and lumbar spine revealed hyperintensities of multiple nerve roots within the cauda equina and T11-12 nerve roots (Figure 5).

**Figure 6:**
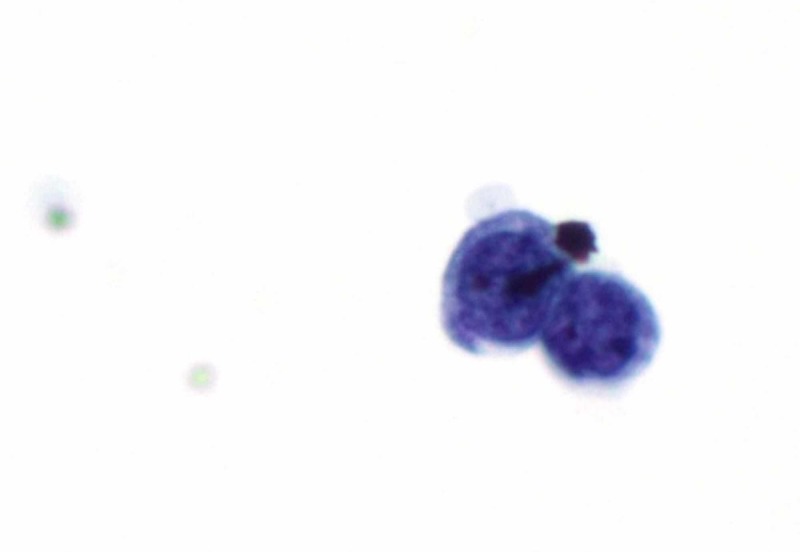
The malignant cells had enlarged, hyperchromatic nuclei, and minimal cytoplasm

The MRI was read as most consistent with arachnoiditis, although a differential of prior infection seqeuale and chronic subarachnoid hemorrhage, less likely LMC was provided. Given her disease history and concerning symptoms, a lumbar puncture (LP) was performed, and the cerebrospinal fluid (CSF) was positive for malignant cells, rendering the diagnosis of LMC (Figure 6).

Surgery was cancelled and her treatment plan was modified to intrathecal chemotherapy and palliative radiation. The patient ultimately succumbed to her disease seven months after initial diagnosis. 

## Discussion

Central nervous system metastasis from head and neck cancers is rare. Additionally, only 2% of LMC arises from head and neck cancers. Diagnosis and treatment decisions for patients with leptomeningeal metastasis from solid tumors vary widely across Europe. Standardization of diagnosis and evaluation tools as well as controlled studies to improve the level of evidence for all therapeutic approaches to LM are required [6]. Major favorable prognostic factors include excellent performance status, absence of serious fixed neurologic deficits, normal CSF flow scans, and absent or responsive systemic tumour. Aggressive therapy for this disorder is often accompanied by a necrotizing leukoencephalopathy which becomes symptomatic months after treatment with radiation and intrathecal methotrexate. As currently available therapies are toxic and provide limited benefits, novel approaches are being studied [7]. Novel diagnostic approaches include the identification of biomarkers that may stratify the risk for developing leptomeningeal metastasis [8]. It is important to keep in mind that the diagnosis can prove elusive and often requires a high index of suspicion to be established, as is highlighted in this case. 

The primary diagnostic techniques in establishing a diagnosis of LMC include CSF cytology and MRI. It is important to note that a negative result on cytology does not exclude the diagnosis of LMC, and additional attempts at obtaining cytology should be made, as after three attempts, the sensitivity of cytology increases to ~90% [9]. In the setting of equivocal imaging, clinical suspicion and decision-making become paramount. Maintaining suspicion for LMC in the setting of new-onset back pain despite inconclusive MRI prompted the lumbar puncture (LP) that identified LMC as the etiology of the patient’s pain. This diagnosis prevented the patient from undergoing surgical resection of the primary tumor, which would not have been curative and would have only exposed the patient to unnecessary morbidity.

## Conclusions

This is a rare occurrence of leptomeningeal spread of SCC of the ethmoid sinus. This diagnosis should be on the differential diagnosis in patients presenting with new neurological deficits.
